# Heterogeneity of Liver Disease in Common Variable Immunodeficiency Disorders

**DOI:** 10.3389/fimmu.2020.00338

**Published:** 2020-02-28

**Authors:** Antonio Pecoraro, Ludovica Crescenzi, Gilda Varricchi, Giancarlo Marone, Giuseppe Spadaro

**Affiliations:** ^1^Department of Translational Medical Sciences, University of Naples Federico II, Naples, Italy; ^2^Center for Basic and Clinical Immunology Research, WAO Center of Excellence, University of Naples Federico II, Naples, Italy; ^3^Department of Public Health, University of Naples Federico II, Naples, Italy; ^4^Monaldi Hospital, Naples, Italy

**Keywords:** primary immuno deficiency, antibody deficiency, common variable immune deficiency, liver disease, nodular regenerative hyperplasia, transient elastography, monogenic immune defects, liver transplant

## Abstract

Common variable immunodeficiency (CVID) is the most frequent primary immunodeficiency (PID) in adulthood and is characterized by severe reduction of immunoglobulin serum levels and impaired antibody production in response to vaccines and pathogens. Beyond the susceptibility to infections, CVID encompasses a wide spectrum of clinical manifestations related to a complex immune dysregulation that also affects liver. Although about 50% CVID patients present persistently deranged liver function, burden, and nature of liver involvement have not been systematically investigated in most cohort studies published in the last decades. Therefore, the prevalence of liver disease in CVID widely varies depending on the study design and the sampling criteria. This review seeks to summarize the evidence about the most relevant causes of liver involvement in CVID, including nodular regenerative hyperplasia (NRH), infections and malignancies. We also describe the clinical features of liver disease in some monogenic forms of PID included in the clinical spectrum of CVID as ICOS, NFKB1, NFKB2, CTLA-4, PI3Kδ pathway, ADA2, and IL21-R genetic defects. Finally, we discuss the clinical applications of the various diagnostic tools and the possible therapeutic approaches for the management of liver involvement in the context of CVID.

## Introduction

Common variable immunodeficiency (CVID) is the most prevalent symptomatic primary immunodeficiency (PID) in adult age and is characterized by marked hypogammaglobulinemia (IgG and IgA, with or without IgM), and impaired antibody production in response to vaccines and pathogens (1, 2). CVID represents an umbrella diagnosis rather than a single disease, probably encompassing multiple genetic disorders, all leading to the failure of B-cell responses. The International Union of Immunological Societies (IUIS) Expert Primary Immunodeficiency Committee (now called Expert Committee on inborn errors of immunity – IEI) redefined in 2009 the acronym CVID as “common variable immunodeficiency disorders,” thus highlighting the heterogeneity of the underlying immune defects (3). During the past 7 years, the increasing spreading of next-generation sequencing (NGS) technologies have fostered the discovery of several genes associated with a CVID-phenotype, *via* both autosomal recessive and dominant inheritance (4, 5). This has progressively blurred the limits between humoral and combined immunodeficiency. Indeed, various genetic defects initially linked to CVID are now recognized as distinct disease entities. However, monogenic forms only account for 2–10% CVID clinical diagnosis (6). The proportion increases to 30% when considering CVID cases with criteria of monogenic form suspicion including early onset, autoimmune/inflammatory manifestations, low B lymphocytes, and/or familial history of hypogammaglobulinemia (7). The pathogenesis is more complex in the remaining cases, probably involving environment, and somatic genetic or epigenetic changes (8). Similarly, several abnormalities in immune cells’ counts and function, in different combinations and in association with specific clinical features, have been described in CVID patients. Among these, the reduction of class-switched memory B cells and/or plasmablasts (9, 10), the expansion of transitional B cells and/or CD21low B cells (11, 12), the reduction of naive T cell and/or Treg cell, and the increase of peripheral blood T_FH_ cells (13, 14), are the most remarkable.

Mirroring this immunologic and genetic heterogeneity, CVID patients may experience a wide spectrum of clinical manifestations during the course of their life, including recurrent bacterial infections (mainly of gastrointestinal and respiratory tracts) and various disorders related to immune dysregulation, such as autoimmunity, granulomata, lymphoid hyperplasia, enteropathy and malignancies (15–17). The cornerstone of CVID treatment is polyvalent human IgG replacement that succeeded, over the past 4 decades, in reducing the burden of infections and improving the prognostic outcome of CVID (18–20). However, immunoglobulin replacement therapy has no proven effectiveness on immune dysregulation-related complications that consequently have become the major cause of death in CVID patients, thus demanding a more in-depth understanding of the underlying pathogenetic mechanisms (21–24).

Immune dysregulation-related complications also involve various segments of the gastrointestinal tract leading to life-threatening complications as protein-energy malnutrition, malabsorption, and gut microbial translocation (25–27). While gut or stomach involvement in CVID has been extensively described and classified by several authors, a more limited evidence is available about prevalence, pathogenesis and prognostic outcome of CVID-related liver disease (28–33). Although up to 50% of CVID patients display a persistent increase of liver enzymes associated with mild hepatomegaly, burden and nature of liver involvement have not been systematically investigated in the majority of CVID cohort studies published in the last 20 years (34, 35). Liver involvement could be defined as a disruption of liver function or portal hemodynamic and may be identified through biochemical, clinical, imaging and histologic diagnostic tools. Liver involvement in CVID is heterogeneous and may rely on immune dysregulation [i.e., nodular regenerative hyperplasia (NRH), lymphocytic infiltration, granulomatous disease], infection (i.e., viral iatrogenic hepatitis, extra-intestinal localization of *Giardia lamblia*) and malignancy (i.e., liver cancer, extra-nodal localization of lymphoid malignancies and metastatic involvement from gastrointestinal tract neoplasms). In a large United States cohort, CVID patients with liver diseases had reduced survival (HR = 2.48), compared with those without this specific complication (23). In particular, liver diseases was the fourth cause of death over a 4-decade interval, accounting for the 8.6% overall mortality. Similarly, in a recent study striking differences in mortality were observed between patients with liver disease and those without, with crude death rate of 28% and 6%, respectively (36). Prevalence information widely varies in the various cohorts (ranging from 9% to 79%) depending on the detection strategy and the sampling methodology (Table 1). In particular, significant heterogeneity exists between the various cohort studies with respect to the outcome variable evaluated to estimate liver impairment (i.e., liver enzyme levels, echographic features, and histopathological changes). Moreover, a large part of prevalence information is derived from cohort studies not primarily conceived to estimate liver involvement. This may result in a significant bias in prevalence data, as incomplete diagnostic assessment could have affected the detection rate of liver alterations in these studies.

**TABLE 1:** Prevalence of liver disease in various cohorts of CVID adult patients.

Study	Year	Sample size	Study type	Prevalence (%)	Outcome variable	Clinical associations
Cunningham-Rundles et al. (37)	1999	248	Retrospective	11.9	Liver dysfunction (including viral hepatitis and primary biliary cholangitis)	NA
Ward et al. (35)	2008	108	Retrospective	43.5	Deranged liver function (i.e., increased liver enzyme levels)	Hepatomegaly, Granuloma, Cytopenias, Lymphocytic enteropathy
Malamut et al. (51)	2008	94	Retrospective	54.2	Increased liver enzyme levels, hepatomegaly and/or signs of portal hypertension	NA
Farmer et al. (38)	2018	205	Retrospective/perspective	9.3	Histologically proved lymphoproliferative liver disease (i.e., NRH and hepatitis)	NA
Slade et al. (147)	2018	116	Cross-sectional	3	Autoimmune liver disease	NA
Azzu et al. (36)	2019	86	retrospective	79	abnormal liver function test profile OR abnormal liver imaging OR abnormal liver histology	Thrombocytopenia, splenomegaly
Crescenzi et al. (40)	2019	77	Cross-sectional	33.8	Liver fibrosis (measured as increased liver stiffness)	Polyclonal lymphoproliferation, enteropathy

Clinical, laboratory and histological signs of liver damage were present in 11.9% subjects of a large US cohort described in 1999 (37). Raised alkaline phosphatase (ALP) levels were observed in 43.5% CVID patients of a 2008 cohort study (35), while histologically proved liver disease was demonstrated in a smaller proportion of subjects in two other studies (9.1% and 9.3%, respectively) (38, 39). Our research group recently reported a liver disease prevalence of 33.8% in a cohort of 77 adult CVID patients in whom liver involvement was assessed through the measurement of liver stiffness by ultrasound-based transient elastography (TE) (40). Finally, 79% CVID patients referred to a United Kingdom Hepatology Center displayed laboratory, imaging and/or histological signs of liver disease (36).

**FIGURE 1:**
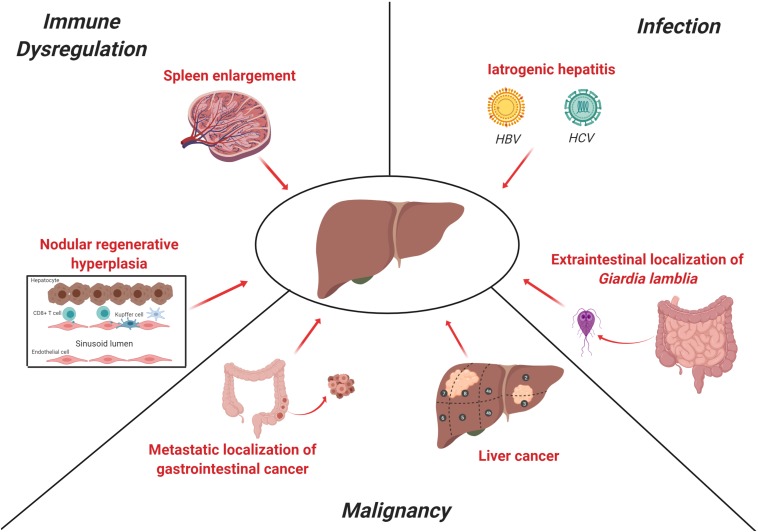
Main causes of liver disease in CVID. The clinical spectrum of CVID includes predisposition to infections, immune-dysregulation-related manifestations (i.e., autoimmunity or lymphocytic infiltration) and malignancies. Liver involvement in CVID may rely on each of these three pathogenetic mechanisms. NRH is the most common histopathologic finding in CVID and is thought to be related, at least in part, to an auto-reactive T-cell intrasinusoidal infiltration, while a subset of patients may present a more severe portal inflammatory infiltration consistent with inferface hepatitis, as observed in autoimmune hepatitis. Splenomegaly is a common clinical feature in CVID patients and contributes to the increase of portal venous pressure, as shown in detail in Figure 2. In past decades, contaminated immunoglobulin preparations were a significant cause of iatrogenic viral hepatitis (i.e., HBV, HCV, CMV, and EBV), while *Giardia lamblia*, which is a common cause of chronic enteritis in CVID, may affect liver as extra-intestinal localization. Finally, liver may be target of both primary and metastatic malignancies. These latter may be the result of both gastrointestinal cancers (i.e., stomach and colon) and hematological malignancies. The figure was created with Biorender.com.

In this review, we will summarize the evidence on epidemiology, pathogenesis, outcome, and treatment of the various forms of liver involvement in CVID (Figure 1). To contribute to better understand and manage CVID-associated liver disease, we will try to depict the features of liver involvement in some monogenic forms of PID included in the clinical spectrum of CVID for which specific defects in immune response pathways have been recently clarified. Finally, we will discuss the clinical applications of the various diagnostic tools employed in detection and monitoring of liver disease.

## Nodular Regenerative Hyperplasia

**FIGURE 2:**
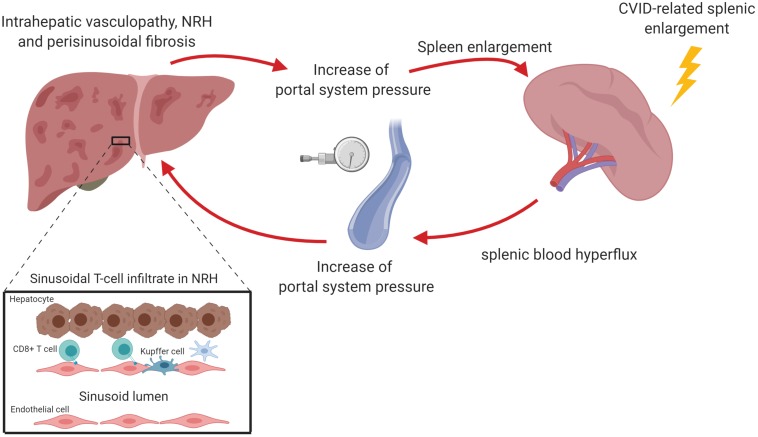
Nodular regenerative hyperplasia (NRH) is the result of an intra-hepatic vasculopathy, leading to the development of hepatocyte nodules that compress surrounding sinusoids, potentially determining perisinusoidal fibrosis. In CVID patients, NRH is associated with a chronic cytotoxic T cell infiltration of liver sinusoidal endothelium. This may cause an alteration of the blood flow through portal system causing the reduction of liver parenchymal perfusion and the increase of portal pressure. The perturbation of portal system flow may also be the result of the hemodynamic changes related to splenomegaly, which causes an increase of splenic venous flow contributing to the increase of portal pressure. The increase of portal pressure could be in turn responsible for a further spleen enlargement. The figure was created with Biorender.com.

Nodular regenerative hyperplasia is generally considered the most typical form of liver involvement in CVID (1). Although frequently described as a disease, NRH is actually a histopathologic picture that is thought to be the result of an intra-hepatic vasculopathy, common to various hepatic diseases, leading to both hepatocyte injury and regeneration (41, 42). This latter would determine the development of hepatocyte nodules that compress surrounding sinusoids, as well as portal and central veins, thus potentially determining perisinusoidal fibrosis (Figure 2) (43, 44). The diagnosis of NRH is challenging due to different interpretations of the histopathologic features and the absence of either symptoms or laboratory abnormalities in most patients. Although nodularity and heterogeneous hepatic parenchyma suggestive of NRH may be detected by magnetic resonance imaging or ultrasound scan, diagnosis has to be histologically confirmed (45, 46). Recently, the revision of the histopathological definition proposed by Wanless in 1990 led to the description of NRH as focal or diffuse appearance of hepatocellular nodules less than 3 mm in diameter detected on both H&E and reticulin staining compressing peripheral sinuses, where perisinusoidal but not septal fibrosis may occur (47, 48).

Post-mortem examination studies reported a prevalence of NRH-related changes in 0.5–2.6% of general population (48, 49). NRH prevalence in CVID widely varies in the various reports, perhaps reflecting different strategies in study design and population sampling (Table 2). Liver biopsy is an invasive procedure that is generally performed only in the presence of clinical and laboratory clues of severe liver damage. This led to a significant underestimate in cohort studies not primarily intended to investigate liver pathology. Resnick et al. reported a NRH incidence lower than 1% over a 4-decade period, in a large perspective cohort study assessing mortality in CVID (23). On the contrary, NRH prevalence was 5% and 12% in 2 studies designed to assess the nature of liver disease in CVID patients with deranged liver function tests (35, 50). Similarly, NRH was detected in 32% patients referred to a Hepatology Center for an active follow-up (36). NRH prevalence is even higher (up to 87%) if we consider only the subset of patients undergoing liver biopsy, namely the only category where NRH diagnosis may be made or excluded with certainty (51).

**TABLE 2:** Prevalence of nodular regenerative Hyperplasia in various cohorts of CVID adult patients.

Study	Year	Sample size	General Prevalence	Prevalence in biopsied patients	Other findings
Ward et al. (35)	2008	108	12% (13/108)	56.5% (13/23)	Clinical association with Hepatomegaly, Granuloma, Cytopenias, Lymphocytic enteropathy
Malamut et al. (51)	2008	94	21.2% (20/94)	86.9% (20/23)	Portal hypertension in 75% of the cases Clinical association with diseases and peripheral lymphocytic abnormalities
Resnick et al. (23)	2012	473	<1% (2/473)	NA	NA
Fuss et al. (50)	2013	261	5.3% (14/261)	NA	64% of NRH patients had elevated hepatic venous pressure gradients (HVPG) consistent with portal hypertension A subset of patients either developed or presented initially with an autoimmune hepatitis-like (AIH-like) Presence of infiltrating T cells producing IFN-γ
Azzu et al. (36)	2019	86	32.5% (28/86)	41.1% (28/68)	A subset of patients had portal hypertension histological cirrhosis, associated with increase in mortality

Although laboratory signs of NRH may be not detectable for decades, the majority of subjects display raised ALP levels with concurrent increase in gamma-glutamyl-transpeptidase (γGT). The most common pattern of ALP derangement in CVID patients with NRH is the gradual increase over years. Otherwise, ALP levels may fluctuate or reach a peak and then return toward normal values (35). When clinical signs are present, these are the result of non-cirrhotic portal hypertension due to sinusoidal compression (50). In the most characterized cohorts of CVID patients with NRH, the most frequent clinical complications were jaundice, hepatomegaly, pruritus, ascites, and oesophageal varices, whereas decreases in neutrophil and platelet counts frequently appeared years after raising of liver enzymes (35, 50, 51). On the other hand, a subset of patients (up to 32%) may present histologically proved cirrhosis with NRH-like changes, a picture associated with higher mortality (hazard ratio = 4.2) (36). Irrespectively of the clinical course of liver disease, CVID patients with NRH are more likely to present immune-dysregulation related complications compared with those without liver involvement. Ward et al. found that NRH was significantly associated with autoimmune cytopenias, polyclonal lymphoproliferation and diffuse granulomatous disease. By contrast, no association was found with organ specific autoimmune conditions, age at onset, age at diagnosis, delay in diagnosis, and duration of immunoglobulin replacement therapy (35).

Intrasinusoidal inflammatory infiltrates represent the most common histopathological finding in CVID patients with NRH (50, 51). Immunohistochemical analysis reveals that infiltrates are mainly composed of CD3^+^ CD8^+^ T cells and very few B cells. Inflammatory infiltrates may co-localize with sinusoidal dilatation and/or small lobular, non-necrotizing, non-fibrosing granulomata (<50% cases). Conversely, albeit rarely, CVID patients may present liver granulomatous lesions in the context of a systemic granulomatous disease and in the absence of NRH (35). A subset of patients exhibit a more severe portal inflammatory infiltration associated with portal vein endotheliitis, bridging necrosis and periportal fibrosis, thus justifying a histological diagnosis of interface hepatitis, as observed in viral or autoimmune hepatitis (50, 51). Rather than the result of a proper autoimmune hepatitis, all these findings could represent an over-representation of the milder inflammatory infiltrate associated with perisinusoidal fibrosis, usually observed in NRH. Consistent with this hypothesis, CVID patients present interface hepatitis in the context of the nodular hepatic parenchymal pattern typical of NRH that is not described in “classical” autoimmune hepatitis (52). Besides, the diagnosis of definite autoimmune hepatitis (AIH) is very difficult to be made in CVID patients. According to the European Association for the Study of the Liver (EASL), both a histologic evidence of moderate to severe interface hepatitis and the positivity of the typical autoantibodies are required to make an AIH diagnosis (53). Indeed, as expected for a severe B-cell defect, CVID patients generally do not have autoantibodies, even in case of overt autoimmune manifestations. On the other hand, NRH *per se* is likely to represent an immune-mediated manifestation. The presence of moderate/severe inflammatory infiltrates could suggest different pathogenetic mechanisms, as well as a possible role for immunosuppressive treatments to arrest the progression of liver damage. Based on this consideration, liver biopsy would represent a pivotal tool to identify the cases of NRH associated with a more significant inflammatory infiltrate and guide the decision to start an immunosuppressive treatment.

Intrasinusoidal T lymphocytes may be involved in the pathogenesis of NRH, as supported by the frequent finding of both portal vein endotheliitis and disruption of the sinusoid lining. Indeed, a significant proportion of NRH patients display apoptotic damage of sinusoidal endothelial cells associated with the presence of CD8^+^ cytotoxic T-cells in liver sinusoids (50, 51). Analysis of liver T cell receptor clonality revealed that intra-sinusoidal T cells specifically targeted sinusoidal endothelial cells. In addition to this, hepatocytes from NRH-patients exhibited overexpression (up to 100-fold) of IFN-γ mRNA compared to controls (50). These findings suggest that NRH may be the result of chronic cytotoxic T cell infiltration of the sinusoidal endothelium. This would be in turn responsible, in association with granulomata, for an alteration of the blood flow through portal system leading to the reduction of liver perfusion.

The perturbation of portal system flow may also be the result of the hemodynamic changes related to splenomegaly, a condition present in about one third of CVID patients. Pulvirenti et al. found that spleen diameter directly correlated with portal vein diameter, suggesting that an increased splenic venous flow related to splenomegaly could contribute to a condition of portal hyper-flux (54). Consistent with this, 25% patients in that cohort had ultrasound signs of portal vein enlargement, even if only 16% of them had portal hypertension. Interestingly, when liver biopsy was performed, the authors reported micronodular transformation and lymphocytic infiltration, signs reminiscent of NRH.

Finally, some of the histopathological changes associated with NRH in CVID patients, as T-lymphocyte infiltrates and/or granulomata, may represent a response to microbial translocation. Microbial translocation is the transfer of commensal microbial products from the intestinal lumen into systemic circulation in the absence of overt bacteremia (55). Although the extent of potential damage to intestinal epithelial barrier in CVID is currently unknown, CVID patients may have increased intestinal permeability resulting from the typical CVID-related enteropathy (28). Of note, the inflammatory changes found in liver often resemble those observed in individuals with chronic inflammation of the gut. In a small group of seven CVID patients with evidence of liver inflammation, intestinal inflammation was found in five cases (56). Consistent with this hypothesis, different studies demonstrated signs of microbial translocation and microbial translocation-related immune activation in CVID patients, as elevated plasma concentration of lipopolysaccharide (LPS), soluble CD14 (sCD14), and soluble CD25 (sCD25) (57–61). However, to the best of our knowledge, no study has already addressed the possible association between impaired intestinal permeability and liver disease in CVID patients.

## Infections

In the past, several cases of iatrogenic viral hepatitis C due to contaminated intravenous immunoglobulin preparations have been reported (62, 63). Several studies reported an increased mortality and morbidity in long-term follow-up of CVID patients iatrogenically infected with HCV, compared to iatrogenic viral hepatitis in general population (64–66). On the other hand, in a small study none of 18 HCV-infected patients developed severe disease nor died because of the infection (67). In more recent CVID cohort studies, a lower prevalence of viral hepatitis was reported, probably reflecting the efficient prevention of viral contamination of blood products achieved in the last three decades. Indeed, the immune defect underlying CVID would not predispose to viral infections, as also suggested by the clinical phenotyping proposed by Chapel et al. in 2008. This classification excluded viral infections, including persistent infection with enterovirus, HBV and HCV, from the clinical phenotyping, as they were not considered part of natural disease progression (68). In the same study, prevalence of hepatitis B and C among 334 CVID patients were about 1% and 6%, respectively.

Similarly, a previous cohort study from Mount Sinai Institute found an overall viral hepatitis cumulative incidence of 6.5% (37). The same research group reported a significantly lower data (1.9% and 1% for HCV and HBV, respectively) 23 years later, thus suggesting that the first cohort probably included a greater proportion of subjects who had received contaminated immunoglobulin preparations (23).

Common variable immunodeficiency patients are not particularly prone to bacterial and/or parasitic infections primarily involving the liver (69). Similarly, the finding of opportunistic or unusual pathogens, such as *Microsporidia* or *Cryptosporidia*, is rare and might suggest investigating for a combined immunodeficiency, characterized by greater degrees of T-cell dysfunction (70). Liver is a possible extra-intestinal localization of *Giardia lamblia*, which is a common cause of chronic enteritis in CVID (70, 71). Therefore, liver involvement should always be ruled out in case of *Giardia* detection from stool or duodenal samples.

## Malignancies

Malignancies are one of the major causes of death in patients with CVID (72–75). Compelling evidence suggests a higher cumulative incidence of malignancy in CVID population (widely ranging from 1.5% to 25.5%), with a peak of incidence between the 4th and 6th decade of life (76). Non-Hodgkin lymphomas are the most common type of malignancy in several cohort studies (23), even though epithelial cancers are associated with a higher mortality ratio and gastric cancer has recently emerged as the leading cause of death in a large multicenter Italian study (22, 77). The pathogenetic mechanisms underlying cancer development in CVID are not completely understood. These might include impairments in various stages of B-cell maturation, primarily yielding lymphoid malignancies, chronic infections and/or low-grade inflammation, which are thought to play a pivotal role in tumor development and growth (78, 79).

In contrast with hematological and gastrointestinal mucosal malignancies, very few data are available about prevalence, distribution and outcome of liver cancers in CVID. Four cases of liver cancer were found in an Italian cohort of 455 adult patients (prevalence 0.95%), corresponding to a Standardized Incidence Ratio (SIR) of 1.9 (95% CI 0.3–5.6) in comparison to the Italian National Cancer Registry (Associazione Italiana Registro Tumori – AIRTUM) data (22). Noteworthy, all four patients died and liver cancer accounted for the 5.1% all death in the cohort. Liver cancer was the fourth cause of death for malignancy after gastric cancer, non-Hodgkin lymphoma and colorectal cancer, with a standardized mortality ratio of 2.9 (95% CI 0.1–5.9) compared to AIRTUM data. Although liver cancer is not prevalent in CVID, liver, as a secondary lymphoid organ, is a frequent extra-nodal localization of non-Hodgkin lymphoma, as well as a common metastatic target of gastrointestinal adenocarcinomas (80–85). Therefore, diagnostic protocols aiming to oncologic surveillance in CVID patients should always encompass clinical, laboratory and imaging assessment of liver to rule out its primary or secondary neoplastic involvement.

## Monogenic Forms of PID in the Clinical Spectrum of CVID

The striking advances in sequencing technologies have fostered the discovery of several genes associated with a CVID-like phenotype (6–8). Actually, mutations in most of them lead to more severe immune dysregulation syndromes compared to CVID, often in association with pronounced T-cell defects. Indeed, mutations affecting these genes are considered to cause separate disease entities rather than a “pure” CVID (5). Here, we discuss the monogenic forms of “CVID-like” PIDs for which liver involvement has been described, seeking to highlight the different features of liver pathology in each form, which could possibly help to drive genetic testing (Table 3).

**TABLE 3:** Genetic, immunological and clinical features in monogenic forms of PID in the clinical spectrum of CVID with liver involvement.

Genetic defect (OMIM)	Effect on protein	Inheritance	Most frequent clinical manifestations	Most frequent Immune phenotype	Liver involvement
ICOS (86–91) (604558)	LOF	AR	Respiratory tract infections Skin infections Opportunistic infections Autoimmunity (i.e., cytopenias and arthritis)	Pan-hypogammaglobulinemia Low/absent naïve B-cells and switched-memory B cells Low T_FH_ cells Low CTLA-4 Low production of Th1/Th2/Th17 cytokines	HHV-6 hepatitis Non-infectious hepatitis (drug-induced?) Hepatomegaly
NFKB1 (92–98) (164011)	LOF (H)	AD	Respiratory tract infections Lymphadenopathy Splenomegaly, GLILD Autoimmune cytopenias Hematological malignancy	Pan-hypogammaglobulinemia Low/absent switched-memory B cells and plasmablasts Normal T-cell phenotype	Increase of liver enzymes Fibrosis and cirrhosis with Liver insufficiency
NFKB2 (99, 100) (164012)	LOF (H)	AD	Respiratory tract infections, Skin infections, Opportunistic infections Lymphocytic organ infiltration Autoimmunity ACTH-deficiency + other endocrinological abnormalities	Pan-hypogammaglobulinemia Low marginal zone and switched-memory B cells Expansion of CD4^+^ T cell with low naïve T cells Low Treg, T_FH_ and TH17 cells	Increase of liver enzymes Lymphocytic infiltration Steatosis Autoimmune hepatitis
CTLA-4 (101–105) (123890)	LOF (H)	AD	Lymphoproliferation, Respiratory tract infections and bronchiectasis Enteropathy Autoimmune cytopenias, Atopic dermatitis Endocrinopathy Neurological disroders EBV-driven lymphomas	Pan-hypogammaglobulinemia Low CD4^+^ T cells with normal Treg cells Low switched-memory B cells Increase of CD21^low^ B cells	Unspecified liver involvement in 12% patients
LRBA (106–110) (606453)	LOF	AR	Autoimmunity cytopenias Enteropathy GLILD Lymphproliferation and lymphocytic infiltration of organs Respiratory and gastrointestinal infections Type 1 Diabetes	Pan-hypogammaglobulinemia Low switched memory B cells and plasmablasts Normal or increased double negative T cells Normal or low Treg cells	Hepatomegaly Autoimmune hepatatis Peri-portal and perisinusoidal fibrosis Granulomata
PI3Kδ pathway (111–118) (602839; 171833; 601728)	GOF of PI3Kδ (APDS1) LOF of PI3Kδ (APDS2) LOF of PTEN (APDS3)	AD	Respiratory tract infections and bronchiectasis Opportunistic and viral infections Lymphoproliferation Autoimmune cytopenia Enteropathy Neurodevelopmental delay	Low IgG and IgA Low naïve and switched-memory B cells Increase of transitional and CD21^low^ B cells Low CD4^+^ naïve T-cells Impaired T-cell response to IL-2	Increase of liver enzymes NRH Sclerosing cholangitis Cirrhosis Cryptosporidium infection
ADA2 (119–122) (607575)	LOF	AR	Recurrent infections Lymphoproliferation Polyarteritis nodosa Livedo reticularis Ischemic/hemorrhagic stroke Bone marrow aplasia Neurological impairment	Hypogammaglobulinemia Low switched-memory B cells Impaired B cell response to CD40-L and IL-21	Increase of liver enzymes NRH with portal sclerosis Vasculitis Hepatomegaly
IL-21R (123–127) (605383)	LOF	AR	Respiratory tract infections and bronchiectasis Opportunistic infections Lymphoproliferation Inflammatory skin disease	Hypogammaglobulinemia Impaired B cell response to IL-21 Variable T cell response to mitogens	Cryptosporidium infection

## ICOS

Inducible co-stimulatory (*ICOS*) deficiency was the first monogenic defect associated with CVID (86). *ICOS* biallelic mutations result in complete loss of protein expression determining low/absent memory B cells and bone marrow plasma cells (87). All *ICOS*-deficient patients present with recurrent respiratory tract infections and autoimmune manifestations (88, 89). The spectrum of disease extended to include liver involvement in 2015, when two patients presenting in early childhood with raised liver enzymes, diarrhea, colitis, and defective clearance of human herpesvirus 6 were described (90). Hepatomegaly and non-infectious hepatitis were found in 20% of a 15-patient *ICOS* deficiency cohort (91). Histological analysis revealed alcoholic steato-hepatitis in one case of non-infectious hepatitis, while pathogenesis remained unclear in the remaining cases, possibly involving drug-induced toxicity.

## NFKB1

Autosomal dominant haploinsufficiency due to heterozygous loss-of-function mutations in nuclear factor kB subunit 1 (*NFKB1*) causes a progressive impairment in the development of immunoglobulin-producing B cells and is now recognized as the most common monogenic cause of CVID (92, 93). Massive lymphadenopathy, splenomegaly and autoimmune cytopenias are the main clinical features of *NFKB1* LOF (94). Liver involvement was described in 37.5% (6/16) patients in a European population study: three patients had persistently raised liver enzymes and three developed liver failure (95). Histologic assessment of liver disease was performed in three patients, showing fibrosis and cirrhosis with no evidence of autoimmune or granulomatous disease. Consistent with this finding, mouse models have suggested a non-immune role for NF-kappa B signaling in patients with liver failure (96). Multiple liver hemangioma and hepatomegaly associated with EBV-driven lymphoproliferation were described by two previous reports (97, 98).

## NFKB2

The clinical phenotype of nuclear factor kB subunit 2 (*NFKB2*) haploinsufficiency is characterized by early-onset antibody deficiency, autoimmunity, lymphocytic organ infiltration and possibly ACTH-deficiency (99). Liver abnormalities reported in literature are parenchymal lymphocytic infiltration (2 patients), mild hepatopathy with elevation of liver enzymes, liver steatosis and histologically proved autoimmune hepatitis (one patient each) (100).

## CTLA-4

Cytotoxic T-lymphocyte antigen 4 (*CTLA4*) is an essential negative immune regulator acting in the suppression of T-cell proliferation and differentiation mediated by regulatory (Treg) cells (101, 102). Heterozygous germline mutations in *CTLA4* cause an immune dysregulation and immunodeficiency syndrome including hypogammaglobulinemia, lymphoproliferation, recurrent respiratory infections and bronchiectasis, enteropathy, autoimmune cytopenias, atopic dermatitis, endocrinopathy, and neurological features (103, 104). The largest multicenter cohort, including 90 affected subjects within 133 *CTLA4* mutation carriers, reports a prevalence of 12% (11/90) of unspecified liver involvement (105). Liver cirrhosis of unknown cause was identified in one patient, while one mutation carrier died for acute liver failure after many years of gastrointestinal disease.

## LRBA

The lipopolysaccharide-responsive and beige-like anchor (*LRBA*) protein deficiency is caused by loss of protein expression, which can be the result of either homozygous or compound heterozygous mutations in *LRBA* (106). *LRBA* plays a pivotal role in *CTLA-4* surface expression, by rescuing endosomal *CTLA-4* from lysosomal degradation. Clinical manifestations of *LRBA* deficiency include early-onset hypogammaglobulinemia, autoimmune manifestations, IBD and recurrent infections (107). The largest cohort study, describing clinical features of *LRBA*-deficiency in 22 subjects, reports hepatomegaly in 24% patients, with three subjects diagnosed with autoimmune hepatitis (108). Histopathological features of liver disease in *LRBA* deficiency have been investigated in a small number of case series, which described lymphocytic (T cell) infiltrates suggestive of autoimmune hepatitis and/or portal and periportal fibrosis associated with bridging cirrhosis and/or granulomata (106, 109, 110).

## PI3Kδ Pathway

Germline mutations leading to hyperactivation of the phosphoinositide 3–kinase δ (*PI3K*δ) pathway cause activated phosphoinositide 3–kinase δ syndrome (APDS) (111). This may be the result of heterozygous gain-of-function mutations in the calalytic subunit of *PI3K*δ *– PIK3CD* (APDS1), heterozygous loss-of-function mutations in the regulatory subunit of *PI3K*δ *– PIK3R1* (APDS2), or loss-of-function mutations in phosphatase and tensin homolog – *PTEN* (APDS3) (112, 113). The most frequent clinical manifestations of APDS are recurrent bacterial and viral infections and non-malignant lymphoproliferation (114). This latter also includes hepatomegaly, typically in association with lymphadenopathy and splenomegaly. In a large series of APDS patients, raised liver enzymes were observed in 27% (9/33) subjects. NRH was the most frequent histological diagnosis (4/5 patients undergoing liver biopsy) and was associated with mildly increased portal pressure, even though clinical signs of portal hypertension were only present in one patient (115). The high prevalence of NRH has possible therapeutic implications, since NRH is known to lead to poor outcome after hematopoietic stem cell transplant (HSCT), which represents the only curative approach to APDS (116). Therefore, the detection of NRH before HSCT may influence the choice of myeloablative preconditioning. Finally, rare cases of cirrhosis and primary sclerosing cholangitis have been reported in APDS cohort studies, while Cryptosporidium species has been isolated in only two cases (117, 118).

## ADA2

Loss-of-function mutations in adenosine deaminase type 2 (*ADA2*) result in an autosomal recessive disease characterized by a heterogeneous clinical picture, probably mirroring the pleiotropic effects of this enzyme (119). Clinical manifestations of deficiency of *ADA2* (DADA2) include hypogammaglobulinemia, recurrent infections, bone marrow aplasia, pure red cell aplasia, neutropenia, liver disease, neurological impairments, and vasculopathy of small- and medium-sized arteries (120, 121). Liver biopsies from DADA2 patients revealed vascular changes characterized by compromised endothelial integrity, endothelial cellular activation and inflammation (120). Elevated liver enzymes and hepatosplenomegaly are the most common liver-related clinical signs (120–122). Histopathologic assessment frequently shows NRH and/or hepatoportal sclerosis, which could potentially lead to portal hypertension and end-stage liver disease (120).

## IL21R

Biallelic loss-of-function mutations in IL21 receptor (*IL21R*) cause a severe syndrome characterized by respiratory tract infections, inflammatory complications and/or opportunistic infections, with elevated mortality in childhood (123). To the best of our knowledge, four *IL21R*-deficient patients with Cryptosporidium-related liver disease have been described (124–126). Of note, one of the first two index patients underwent liver transplantation (LT) before both the underlying PID and the Cryptosporidium infection had been recognized (124). He died shortly after the procedure due to multiorgan failure. Although no clinical association between *IL21R* deficiency and liver malignancy has been described in humans, an interesting mice model demonstrated that *IL21R* signaling deficiency might promote hepatocellular carcinoma (HCC) growth. Interestingly, Zheng et al. reported that *IL21R* deletion reduced T cells infiltration, activation and functions while increased the infiltration of myeloid-derived suppressor cells that enhanced HCC growth (127). If confirmed in human studies, this finding could affect long-term follow-up strategies of liver involvement in *IL21R*-deficient patients.

## Diagnostic Work-Up

The laboratory panel to assess liver impairment in CVID includes full blood count, liver function tests – LFTs (i.e., AST, ALT, ALP, γGT, total protein, and albumin) and clotting profile (i.e., INR, APTT, fibrinogen). Given the heterogeneity of liver disease, as well as the number of drugs (notably immunosuppressant) and the wide range of non-primarily hepatic complications that may possibly affect liver function in the context of CVID, we believe that this profile should be repeated every 4–6 months, also in asymptomatic patients (15–17, 128). In addition, we perform a wide screening for hepatitis viruses based on nucleic acids detection methods, at the time of diagnosis and at 1-year intervals, due to the virtual risk of viral contamination of immunoglobulin preparations (62–64). Actually, this timing reflect our own clinical practice as no specific guidelines or clinical consensus have been defined. ALP is the most commonly elevated liver enzyme in CVID and its increase is up to twofold above the upper limit on overage (34, 50). Ward et al. identified three distinct patterns of ALP derangement in CVID patients with abnormal LFTs, consisting in progressive elevation, fluctuating increases and transient increase (35). In a cohort of CVID patients with NRH, ALP raise was first observed 6–10 years after the time of CVID diagnosis, while the increase in ALT/AST ratio occurred over the same period but at a lesser degree (50). Noteworthy, elevation of ALP may also be caused by osteomalacia as a result of enteropathy or granulomatous disease, which are common complications in CVID (34, 35).

Ultrasonography, computed tomography scan (CT), or magnetic resonance imaging (MRI) may be employed to detect structural changes (as signs of NRH, cirrhosis and/or portal hypertension), estimate hepatomegaly and/or splenomegaly, and rule out primary or secondary malignant involvement (34). Due to low costs, wide availability, and non-invasiveness, we suggest performing ultrasonography with Doppler-evaluation as first-line liver imaging in all CVID patients, while CT and MRI may be prescribed, even at the suggestion of the Radiologist or the Hepatologist, to better characterize abnormalities detected by US.

Results of CT and MRI scans revealed portal vein dilatation and collateral vessel formation in 50% CVID patients with NRH described by Fuss et al. (50), while abnormal liver imaging was present in 77% of CVID patients started to an active hepatology follow-up reported by a more recent United Kingdom cohort study (36). On the other hand, histopathological changes consistent with NRH were found in a subset of patients with normal liver imaging, who had undergone liver biopsy because of abnormal LFTs. This suggests that liver biopsy should be considered in all patients with persistently abnormal LFTs (36).

In the last decade, ultrasound-based TE has been increasingly used to improve the detection of the progression of liver damage in the context of chronic HCV-disease (129). TE allows estimating the degree of liver fibrosis through the assessment of liver stiffness and depends on vibration generating machine to apply vibrations to the liver and then obtain the propagation velocity of shear wave (130). We recently investigated liver involvement in a cohort of CVID adult patients by means of ultrasound based TE, finding that 33.8% patients presented increased liver stiffness values ranging from moderate fibrosis to cirrhosis (40). Interestingly, TE values were correlated with ALP and γGT values, spleen longitudinal diameter and peripheral blood counts. Moreover, liver stiffness was higher in patients with polyclonal lymphoproliferation and/or enteropathy, and subjects harboring both these complication showed a significantly increased risk (OR: 7.14) of having increased TE values. Therefore, given its non-invasive nature, the limited costs and the crucial information provided, we suggest repeating ultrasound-based TE, as well as canonical ultrasound scan with Doppler evaluation, every 12 months, also in asymptomatic CVID patients. On the other hand, although TE assessment allow to reliably estimating fibrotic changes of liver parenchyma, it does not provide information about the extent and the trend of stiffness variations related to the differet underlying pathogenetic process (i.e., inflammation, granulomatous disease, and lymphocytic infiltration). Further studies, evaluating the concordance between stiffness values and liver histological changes, are required to assess the role of elastography in the evaluation and management of liver involvement CVID.

While the spreading of TE and the systematic use of the various imaging techniques may determine a reduced overall need for liver biopsy, histological analysis of the hepatic parenchyma remains the only tool to ascertain the etiopathogenetic nature of liver damage and confidently estimate its outcome (131). On the other hand, liver biopsy is an invasive procedure, associated with an estimated morbidity and mortality rate in general population of 3% and 0.01%, respectively, with bleeding being the most relevant cause (132). In the context of CVID, this procedure may be theoretically burdened by an additional infectious risk due to the underlying immune defect. Moreover, liver biopsy provides only a very small part of the whole organ, which could be not representative for the degree of the pathological status of the remaining parenchyma, due to the heterogeneity usually observed in liver injury distribution (133). In general, indications for liver biopsy fall into two groups: establishing a diagnosis (including the assessment of the predominant cause of liver injury if more than one is present) and staging/grading liver damage (134). Indeed, in both cases the result of histological assessment may modify the therapeutic management, offering the patient personalized therapeutic options. We suggest that liver biopsy should be considered for CVID patients with a significant (more than twofold the upper limit of the range) unexplained increase of one or more liver enzymes, lasting more than 6 months. The association with pathological liver stiffness values and/or imaging findings of uncertain interpretation strengthens this recommendation. However, we believe that the decision to perform a biopsy and its timing should rely on both the pathological processes being suspected and the possibility of a potential therapeutic intervention.

## Therapeutic Perspectives: Liver Transplantation and HSCT

Irrespectively of the ethiopathogenesis and despite the adequate treatment of complications (i.e., portal hypertension, jaundice, and oesophageal varices), chronic liver inflammation may cause a progressive disruption of liver function that is not improved by immunoglobulin replacement therapy. Moreover, there are no available medical treatments to arrest the histopathologic progression of NRH, which is the most common form of liver involvement in CVID and is complicated, in a subset of patients, by portal hypertension or overt hepatic cirrhosis with end-stage liver disease (44–47). In these cases, LT is the only therapeutic approach that has the potential to provide a long-term survival advantage (135). According to the European Association for the Study of the Liver (EASL), LT should be considered in any patient with end-stage liver disease, in whom the LT would extend life expectancy beyond what the natural history of underlying liver disease would predict or in whom LT is likely to improve the quality of life (136).

On the other hand, the theoretical increase of infectious and neoplastic risk related to the long-term concomitant immunosuppressive therapy has historically determined a reluctance to perform LT in CVID patients. In the last decade, a growing number of reports described the outcome of LT performed in adult and pediatric CVID patients with viral hepatitis or NRH (137–143).

A retrospective Norwegian cohort study reported five CVID patients transplanted over a 20-year period (137). The first patient, transplanted in 1993 for HCV-related disease, died because of sepsis combined with a debilitating *Cryptosporidium parvum* infection and cytomegalovirus pneumonitis, whereas, the 4 patients transplanted between 2009 and 2013 for definite or probable NRH, were alive at the time of publication, with a median survival of 5 years. This different outcome is likely to be related to the changes in immunosuppressive drug regimens from the 1990s to 2009–2013, which consist in the decrease of the glucocorticoid doses. More recently, Azzu et al. described four CVID patients undergoing LT for end-stage liver failure, in whom histological examination revealed NRH-like changes (138). In three subjects out of four, post-transplant course was complicated by multiple infectious complications (including *Pneumocystis jiroveci* pneumonia, toxoplasmosis, neuro-aspergillosis, and CMV proctitis), early recurrent disease, and in one patient, death due to malignancy within 3 years of transplantation. Noteworthy, histological examination showed NRH changes and cholestasis in all three patients undergoing post-transplant biopsy, as already previously described in non-immunodeficiency subjects (144). After a revision of literature data, including 18 patients, the authors found that CVID patients undergoing LT had a higher mortality compared to LT in general population, with only 55% subjects alive after 3-5 years of post-transplant follow-up (138). Moreover, CVID patients undergoing LT due to CVID-related liver disease (namely NRH) exhibited a worse 5-year survival compared to CVID patients who received LT for any cause (mainly chronic viral hepatitis) (138). This probably reflects the fact that the latter subset of patients presented a lower incidence of immune dysregulation-related complications, which are associated with worse long-term survival and higher risk of recurrent of disease in the graft. In this subset of patients, there could be a theoretical benefit of combined hematopoietic stem cell and LT.

Hematopoietic stem cell transplantation (HSCT) could theoretically prevent the development of liver disease or arrest progression in subjects with established liver disease, with a significant improvement of long-term outcome. HSCT is the standard of care of a broad group of severe combined primary immunodeficiencies primarily affecting T-cell functions, as well as of other complex primary immunodeficiencies (i.e., chronic granulomatous disease, Hyper-IgE syndrome, Wiskott-Aldrich syndrome, etc.) (145). The growing evidence of both T-cell defects and poor outcome in the subset of patients with marked immune dysregulation, have progressively fostered the interest in HSCT for the treatment of CVID. In the largest multicenter study collecting data of CVID patients undergoing HSCT, overall survival rate was 48% after 2 years, with immune dysregulation (i.e., autoimmune cytopenias, enteropathy, generalized granulomatous disease) and hematological malignancies being the major indications to transplantation (146). The major causes of death were treatment-refractory graft-versus host disease (GvHD), poor immune reconstitution and infectious complications. On the other hand, IgRT was stopped in 50% and the condition constituting the indication for HSCT resolved in 92% of surviving patients, thus suggesting that this therapeutic approach could be beneficial in selected patients. Indeed, the definition of criteria for both patient selection and transplantation timing, as well as the refinement of the procedure protocol, are urgently needed to improve the outcome of CVID patients undergoing HSCT.

## Conclusion

Although more than 50% CVID patients exhibit clinical or biochemical signs of liver derangement, burden and nature of liver involvement have not been systematically investigated by the major part of CVID cohort studies published in last decades. This lack of evidence lead to the absence of indications or guidelines concerning diagnosis, investigation and management of CVID-associated liver disease in clinical practice. Moreover, the striking advances in sequencing technologies has fostered the discovery of several genes associated with monogenic CVID disorders for which specific liver alterations have been described. We sought to provide a comprehensive overview of both the different causes of liver involvement in CVID and the various monogenic defects associated with liver disease, in order to facilitate the Clinical Immunologist in the diagnostic and therapeutic approaches. The clinical spectrum of CVID includes predisposition to infections, immune-dysregulation-related manifestations (i.e., autoimmunity or lymphocytic infiltration) and malignancies. Liver involvement in CVID may rely on each of these three pathogenetic mechanisms NRH is the most common liver histopathological change observed in CVID patients and is thought to be the result of an intra-hepatic vasculopathy, leading to the development of hepatocyte nodules that compress surrounding sinusoids, potentially determining perisinusoidal fibrosis. Therefore, NRH has the potential to determine a significant alteration of the blood flow through portal system, thus promoting the development of portal hypertension. Infections could either primarily (as in the case of iatrogenic viral hepatitis due to contaminated immunoglobulin preparations in past decades) or secondarily (extra-intestinal localization of parasites) affect liver. Similarly, liver may be target of both primary and metastatic malignancies. Given the heterogeneity of liver disease and the possible impact on long term outcome, each CVID patient should be screened for a possible liver impairment through biochemical (i.e., AST, ALT, ALP, γGT, and total protein and albumin) and morphological (i.e., ultrasonography, TE, and eventually CT or MRI) assessments that should be performed at regular intervals. These diagnostic tools may help to timely identify liver involvement, monitor its progression and select patients eligible to liver biopsy. Despite early detection and adequate treatment of complications, chronic liver damage may progress toward an end-stage disease. In these cases, LT and hematopoietic stem cell transplantation are the only therapeutic approaches that have the potential to provide a long-term survival advantage, even though serious warnings still subsist about the outcome of these procedures in CVID patients. Indeed, compelling evidence concerning the applications of these therapeutic options are urgently needed.

## Author Contributions

AP, LC, and GS conceived the work and selected the data sources. AP and LC wrote the manuscript and realized the figures. All authors revised the data sources and manuscript text.

## Conflict of Interest

The authors declare that the research was conducted in the absence of any commercial or financial relationships that could be construed as a potential conflict of interest.
